# Runx2 contributes to murine *Col10a1* gene regulation through direct interaction with its cis-enhancer

**DOI:** 10.1002/jbmr.504

**Published:** 2011-09

**Authors:** Feifei Li, Yaojuan Lu, Ming Ding, Dobrawa Napierala, Sam Abbassi, Yuqing Chen, Xiangyun Duan, Siying Wang, Brendan Lee, Qiping Zheng

**Affiliations:** 1 Department of Anatomy and Cell Biology, Rush University Medical CenterChicago, IL, USA; 2 Department of Pathophysiology, Anhui Medical UniversityHefei, China; 3 Institute of Oral Health Research, University of Alabama at BirminghamBirmingham, AL, USA; 4 Department of Molecular and Human Genetics, Baylor College of MedicineHouston, TX, USA; 5 Howard Hughes Medical Institute, Baylor College of MedicineHouston, TX, USA; 6 The Student Inquiry and Research (SIR) Program, Illinois Mathematics and Science AcademyAurora, IL, USA

**Keywords:** *Col10a1*, hypertrophic chondrocytes, cis-enhancer, Runx2, transgenic studies

## Abstract

We have recently shown that a 150-bp *Col10a1* distal promoter (−4296 to −4147 bp) is sufficient to direct hypertrophic chondrocyte-specific reporter (*LacZ*) expression in vivo. More recently, through detailed sequence analysis we identified two putative tandem-repeat Runx2 binding sites within the 3′-end of this 150-bp region (TGTGG*G*-TGTGG*C*, −4187 to −4176 bp). Candidate electrophoretic mobility shift assay (EMSA), chromatin immunoprecipitation, and transfection studies demonstrate that these putative Runx2 sites bind Runx2 and mediate upregulated *Col10a1*/reporter activity in vitro. Transgenic studies using the 5′-sequence without Runx2 sites were not able to drive the cell-specific *LacZ* reporter activity, suggesting the in vivo requirement of the Runx2 sites located in the 3′-end in mediating *Col10a1*/reporter expression. Indeed, mutating the Runx2 sites in the context of the 150-bp promoter abolishes its capacity to drive hypertrophic chondrocyte-specific reporter expression in transgenic mice. We have also generated multiple transgenic mouse lines using only the 3′-sequence containing the Runx2 sites to drive the *LacZ* gene. Interestingly, no hypertrophic chondrocyte-specific blue staining was observed in these transgenic mice. Together, our data support that Runx2 directly interacts with murine *Col10a1* cis-enhancer. This interaction is required but not sufficient for cell-specific *Col10a1* promoter activity in vivo. Additional cooperative/repressive elements within the 5′- or 3′-sequences of this 150-bp promoter are needed to work with Runx2 together to mediate cell-specific *Col10a1* expression. Further delineation of these elements/factors has the potential to identify novel therapeutic targets for multiple skeletal disorders, including osteoarthritis, that show abnormal *Col10a1* expression and altered chondrocyte maturation. © 2011 American Society for Bone and Mineral Research

## Introduction

Chondrocyte maturation is the terminal phase of chondrocyte differentiation, a critical stage of endochondral ossification linking both bone and cartilage formation during skeletal development. The type X collagen gene (*Col10a1*), a cell-specific marker of hypertrophic chondrocytes, is involved in controlling the later stages of endochondral bone formation.^(1, 2)^ It has been shown that type X collagen plays a role in normal distribution of matrix vesicles and proteoglycans within the growth plate matrix. Collagen type X deficiency may impact the supporting properties of the growth plate and the mineralization process, resulting in abnormal trabecular bone.^(3)^ Regulatory dysfunctions of *Col10a1* have been closely linked to altered chondrocyte maturation that has been observed in multiple skeletal dysplasias, bone repair and regeneration, as well as in the pathogenesis of osteoarthritis.^4^–^9^

Mutations in *COL10A1* are known to be responsible for two similar human skeletal dysplasias: spondylometaphyseal dysplasia and metaphyseal chondrodysplasia, Schmid type.^(4, 5)^ Schmid metaphyseal chondrodysplasia (SMCD) is characterized by short stature, bowed legs, and coxa vara, suggesting defective long-bone development. It has also been reported that *Col10a1* null mice have disturbed mineralization, altered hematopoiesis, and growth plate compressions that partially resemble SMCD.^(3)^ During fracture healing, endochondral ossification occurs in the fracture callus. Type X collagen synthesis is observed in the cartilaginous callus, which is composed of hypertrophic and degenerative chondrocytes, suggesting increased vascularity and matrix mineralization during fracture repair.^(10)^ As to the correlation of *COL10A1* expression and chondrocyte maturation with osteoarthritis, previous studies have reported the upregulation of *COL10A1* and enhanced chondrocyte hypertrophy in human osteoarthritic cartilage.^(11,12)^ It was also suggested that upon osteoarthritis progression, factors that constrain articular chondrocyte maturation are relieved. These articular chondrocytes achieve a mature phenotype that is characterized by expression of hypertrophic hallmarks, including *Col10a1*.^(13, 14)^

These findings have clearly demonstrated that physiological distribution of type X collagen during chondrocyte hypertrophy is essential for endochondral bone formation in skeletal development, whereas altered *Col10a1* expression is observed in multiple skeletal disorders associated with abnormal chondrocyte maturation. Therefore, understanding the molecular regulation of cell-specific *Col10a1* expression is essential to understanding the basic mechanisms of bone growth as well as the pathogenesis of *Col10a1-*related skeletal diseases.

We have a long-standing interest and have contributed to the understanding of mouse type X collagen gene regulation.^(15, 16)^ Previously, we have shown that a 4-kb *Col10a1* proximal promoter containing Runx2 binding sites was responsible for weak reporter (*LacZ*) expression selectively in lower hypertrophic chondrocytes of transgenic mice.^(15)^ We have recently demonstrated that a 150-bp *Col10a1* distal promoter contains the major cis-enhancer that is sufficient to mediate its hypertrophic chondrocyte-specific reporter (*LacZ*) expression in vivo.^(16)^ In this study, we report further characterization of this 150-bp *Col10a1* distal promoter using combination of in vitro biochemical, cell transfection, and in vivo transgenic approaches as previously described.^(15, 16)^ Our results suggest that Runx2 contributes to regulation of cell-specific *Col10a1* expression through direct interaction with its cis-enhancer containing the two tandem repeat Runx2 binding sites.

## Materials and Methods

### Electrophoretic mobility shift assay

**Figure 1:**
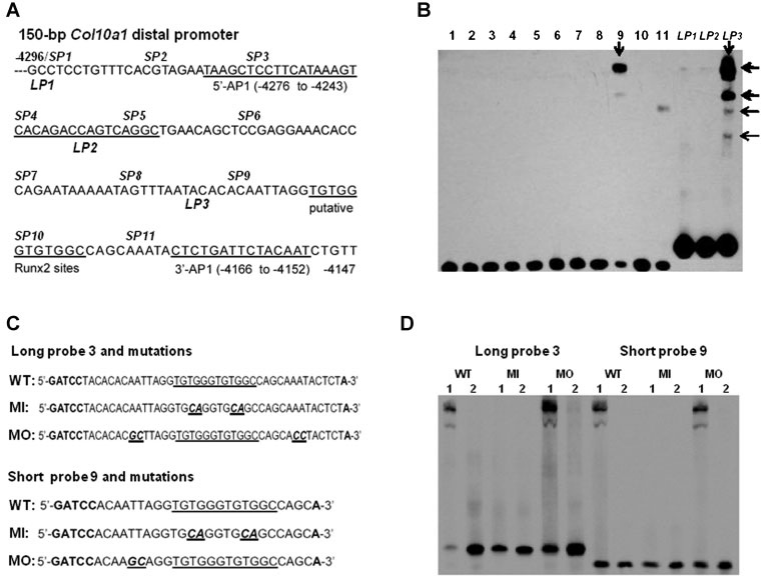
Putative Runx2 sites within 3′prime of the 150-bp *Col10a1* promoter. (*A*) Positions of 11 consecutive short DNA oligos (25 bases each, *SP1-11*) and three long DNA oligos (∼40 bases each, *LP1-3*) within the 150-bp *Col10a1* distal promoter (−4296 to −4147 bp) were as indicated (see also Table 1). The previously reported two AP-1 sites^(16)^ and the putative tandem-repeat Runx2 binding sites (−4187 to −4176 bp) were underlined. *SP* = short probe; *LP* = long probe. (*B*) EMSA assay showed that *SP9* forms specific binding complexes with hypertrophic MCT cell nuclear extracts (lane 9, arrow). Weak signal was also observed with SP11 (lane 11). Similar migration pattern but stronger signal intensity was observed with *LP3* (lane *LP3*, arrows). The sequence of *LP3* (−4201 to −4163 bp) and *SP9* (−4296 to −4274 bp) was shown in (*A*) and in Table 1. Bottom signals correspond to free probes. (*C*) Forward oligo sequences of *LP3* and *SP9* are shown. The putative Runx2 core binding sites (underlined) as well as mutations inside (“CA”) or outside (“GC”, or “CC”) of the core binding sequence are as highlighted (bold and italic). “GATCC” and “A” are BamHI and BglII adaptor sequence. WT = wild type; MI = mutated inside; MO = mutated outside. (*D*) EMSA assays with probes *LP3*, *SP9*, and their mutant forms of oligomers were performed. Probes *LP3* and *SP9* form similar binding complexes with hypertrophic MCT cell nuclear extracts as seen in (*B*) (WT, lane 1). When the putative Runx2 core binding sites were mutated, no DNA/protein complexes were observed (MI, lane 1). However, mutations outside of the core sequence did not abolish the DNA/protein complexes (MO, lane 1). No binding complexes formed when competitive DNA oligos without biotin modification were used (lane 2). Bottom signals show free probes.

Electrophoretic mobility shift assays (EMSAs) were performed using hypertrophic MCT cell nuclear extracts and a series of annealed DNA oligonucleotides (oligos or probes) derived from the cell-specific 150-bp *Col10a1* distal promoter.^(16)^ The nuclear extracts from hypertrophic MCT cells were prepared as previously described.^(15, 16)^ DNA oligos that cover the entire 150-bp *Col10a1* distal promoter were designed and commercially synthesized by IDT Technologies (Coralville, IA, USA). These oligos include 11 short consecutive DNA oligos (25 bases with 12–13 bases of overlapping sequence, *SP1-11*) and three long DNA oligos (∼40 bases, *LP1-3*) as indicated (Fig. 1*A*; Table 1). The mutant derivatives for short probe 9 (−4197 to −4171 bp) and long probe 3 (−4201 to −4163 bp) were also synthesized. Mutations inside or outside of the putative Runx2 binding core sequence (−4187 to −4176 bp) were introduced and as illustrated (Fig. 1*C*; Table 1). The forward oligos were synthesized with or without 5′-biotin modification. Both forward and reverse oligos were synthesized with a BamHI and a BglII adaptor at either 5′- or 3′-end for cloning (Table 1). Runx2 (sc-8566) and Hif1α (sc-13515) antibodies were purchased from Santa Cruz Biotechnology, Inc. (Santa Cruz, CA, USA). Control mouse immunoglobulin G (IgG) was purchased from Invitrogen.

**Table 1:** Oligo Sequences (Forward Only) Used for EMSA Studies

Oligo names	Sequences
*SP1-Forward*	5′-***GATCC***GCCTCCTGTTTCACGTAGAATAAGC***A***-3′ (−4296 to −4272 bp)
*SP2-Forward*	5′-***GATCC***CGTAGAATAAGCTCCTTCATAAAGT***A***-3′ (−4283 to −4259 bp)
*SP3-Forward*	5′-***GATCC***TCCTTCATAAAGTCACAGACCAGTC***A***-3′ (−4271 to −4247 bp)
*SP4-Forward*	5′-***GATCC***CACAGACCAGTCAGGCTGAACAGCT***A***-3′ (−4258 to −4234 bp)
*SP5-Forward*	5′-***GATCC***AGGCTGAACAGCTCCGAGGAAACAC***A***-3′ (−4246 to −4222 bp)
*SP6-Forward*	5′-***GATCC***CCGAGGAAACACCCAGAATAAAAAT***A***-3′ (−4233 to −4209 bp)
*SP7-Forward*	5′-***GATCC***CCAGAATAAAAATAGTTTAATACAC***A***-3′ (−4221 to −4197 bp)
*SP8-Forward*	5′-***GATCC***AGTTTAATACACACAATTAGGTGTG***A***-3′ (−4208 to −4184 bp)
*SP9-Forward*	5′-***GATCC***ACAATTAGGTGTGGGTGTGGCCAGC***A***-3′ (−4196 to −4172 bp)
*SP10-Forward*	5′-***GATCC***GGTGTGGCCAGCAAATACTCTGATT***A***-3′ (−4183 to −4159 bp)
*SP11-Forward*	5′-***GATCC***AAATACTCTGATTCTACAATCTGTT***A***-3′ (−4171 to −4147 bp)
*LP1-Forward*	5′-***GATCC***AGGGTTGGGCCTCCTGTTTCACGTAGAATAAGCTCCTTC***A***-3′ (−4304 to −4266 bp)
*LP2-Forward*	5′-***GATCC***CAGTCAGGCTGAACAGCTCCGAGGAAACACCCAGAAT***A***-3′ (−4251 to −4215 bp)
*LP3-Forward*	5′-***GATCC***TACACACAATTAGGTGTGGGTGTGGCCAGCAAATACTCT***A***-3′ (−4201 to −4163 bp)

*SP* = short probe; *LP* = long probe; Box = putative Runx2 binding sites; *GATCC*/*A* = BamH I/Bgl II adapter.

We performed EMSA using LightShift Chemiluminescent EMSA kit (Pierce, Rockford, IL, USA) with modifications. Briefly, 5′-biotin-labeled forward oligos were annealed to their complementary oligos to obtain the double-stranded probe. Twenty femtomoles (20 fmol) of biotin-labeled probes were then incubated with 5 µg of the MCT cell nuclear extracts at room temperature for 20 minutes. The total reaction volume was 20 µL, which included 1× binding buffer with addition of glycerol (2.5%), MgCl_2_ (5 mM), poly(dI–dC) (50 ng/µL), and NP-40 (0.05%) as provided (Pierce; Catalog number 20148). No biotin-labeled annealed oligos were used for competition. These oligos as well as Runx2 or the control antibody (anti-Hif1α) were incubated 20 minutes before the binding reaction. Binding samples were subjected to electrophoresis in a 6% native polyacrylamide gel and run at room temperature in 0.5× TBE at 20 mA for 40 minutes. The binding DNA/protein complexes were then transferred to positively charged Nylon membrane (Thermo Scientific; Cat. No. 77016, Rockford, IL, USA) in a mini PROTEAN Tetra cell (BioRad, Hercules, CA, USA) and subjected to ultraviolet (UV) cross-link using the Spectrolinker XL-1000 UV crosslinker (Spectronics Corporation, Westbury, NY, USA). Detection of the biotin-labeled DNA was performed according to the manufacturer's protocol (Pierce; Catalog number 89880) using the stabilized streptavidin–horseradish peroxidase (HRP) conjugate and the chemiluminescent substrate module. Visualization of the binding complexes was to expose the membrane to X-ray film or the CCD camera of an AlphaImager (Alpha Innotech) with adjusted time to obtain ideal signal.

### Chromatin immunoprecipitation assay

**Figure 2:**
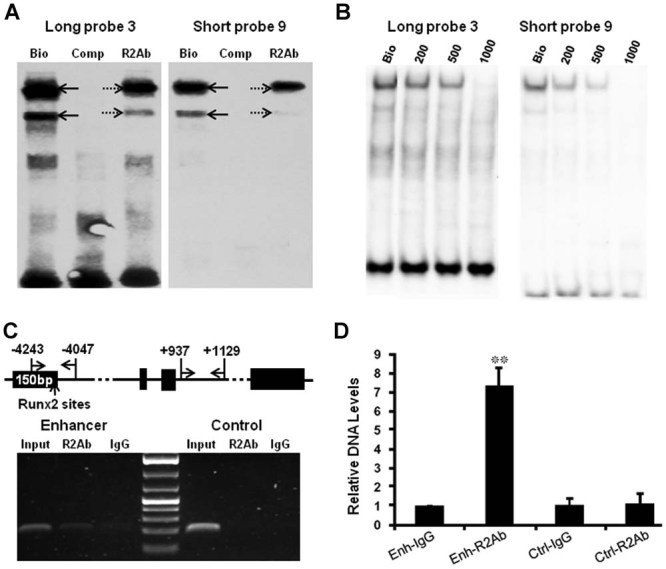
Runx2 binds to the putative tandem-repeat Runx2 binding sites. (*A*) Candidate EMSA assay using Runx2 antibody showed that the signal intensity of the two major DNA/protein complexes decreased when Runx2 antibody was used (lanes “R2Ab,” dashed arrows) compared to the ones using only biotin-labeled probes *LP3* and *SP9* (lanes “Bio,” black arrows). No binding complexes formed using competitive DNA oligos (“Comp” lanes). Bottom signals show free probe *LP3* (left). Free probe *SP9* runs out of the gel (right). Bio = biotin-labeled probes; Comp = probes without biotin modification; R2Ab = Runx2 antibody. (*B*) Candidate EMSA assay was also performed using a diluted Runx2 antibody series. Signal intensity decreased with 200 ng of Runx2 antibody (“200” lanes). Meanwhile, 500 ng of Runx2 antibody significantly inhibited, whereas 1000ng of Runx2 antibody completely abolished, formation of the DNA/protein complexes. Bottom signals show free probes *LP3* and *SP9*. (*C*). ChIP experiment was performed using MCT cells and Runx2 antibody (R2Ab). Position of the primers flanking the enhancer and control sequence was illustrated (top, arrows). Semiquantitative PCR showed clear amplicon of the target enhancer sequence precipitated by Runx2 antibody but only faint band by control IgG (bottom, left), whereas the control sequence was barely detectable from DNA samples that use either Runx2 antibody or control IgG (bottom, right). Both enhancer and control sequences were amplified from the input samples. (*D*) Real-time PCR was performed using the same input sample and DNAs precipitated with Runx2 antibody or control IgG. The enhancer sequence was significantly enriched (7.44-fold, *p* = 0.009) by Runx2 antibody compared to DNA precipitated by control IgG, whereas no enrichment of control sequence was obtained (*p* = 0.878) by Runx2 antibody.

Chromatin immunoprecipitation (ChIP) analysis using hypertrophic MCT cells and Runx2 antibody (sc-8566; Santa Cruz Biotechnology, Santa Cruz, CA, USA) was based on the protocol provided by the manufacturer (Pierce Agarose ChIP Kit, Catalog #26156; Thermo Scientific) and published protocols with modifications.^(17)^ Briefly, hypertrophic MCT cells were incubated at 37°C for 3 days and were cross-linked by 1% formaldehyde for 10 minutes at room temperature followed by glycine treatment to stop the cross-linking for 5 minutes. Cell lysis and micrococcal nuclease digestion was optimized to shear chromatin DNA to primarily 200 to 400 bp. Ten percent (10%) of total precleared chromatin (input sample) was used for immunoprecipitation with 5 µg each of the Runx2 antibody or control IgG using the columns provided by the kit. Proteinase K digestion of the cross-linked protein, reverse cross-linking, and DNA purification were also performed according to manufacturer-provided protocol. The primers used for semiquantitative PCR and real-time PCR amplification were synthesized by IDT Technologies. Primers flanking the *Col10a1* distal promoter containing the tandem-repeat Runx2 binding sites are: 5′-CTG AAC AGC TCC GAG GAA AC-3′ (forward), 5′-TGG ATA TTC AGC CCT TTT GG-3′ (reverse). The control primers are within *Col10a1* intron II, which does not contain Runx2 binding sites: 5′-AAT GAT GCA TGG AAA CGA CA-3′ (forward): 5′-GCC TAT GCA ATT GTT TTT AGC TT-3′ (reverse, Fig. 2). Semiquantitative and real-time PCR was performed using immunoprecipitated DNA elutes as described in the following section.

### Semiquantitative PCR and real-time PCR

Two microliters (µL) of the immunoprecipitated elutes from Runx2 antibody or control IgG and diluted input control were used as template for semiquantitative and real-time PCR. For semiquantitative PCR, we optimized the PCR condition to amplify the target (promoter or enhancer sequence containing the Runx2 sites) and the control (intron II without Runx2 sites) sequences using the specific primers as described in the previous section. Following 30 cycles of amplification, the PCR products were run on a 1.5% agarose gel and analyzed by ethidium bromide staining. For real-time PCR, the same amount of DNA templates and primers were used for PCR amplification using the MyiQ Single Color Real-Time PCR Detection System and SYBR Green real-time PCR master mix (Bio-Rad). The mean threshold cycle number (Ct) values indicating relative DNA levels of target or control sequences were normalized to input samples and were analyzed using 2^−ΔΔ^Ct and Student's *t* test approaches.^(15)^, 17–19 Data is collected from multiple runs of real-time PCR with duplicate templates. *p* < 0.05 implies significant enrichment of target or control sequences using Runx2 antibody or control IgG.

### Cell culture and transfection studies

**Figure 3:**
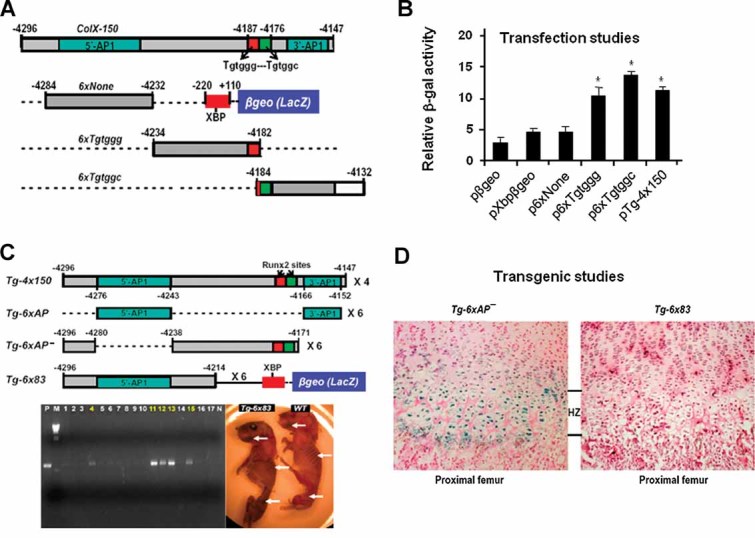
Reporter analysis using 5′-sequence of the 150-bp promoter. (*A*) The transgenic construct (*Tg-4x150*) and reporter constructs containing *Col10a1* basal promoter (*pXbpβgeo*), six copies of the cis elements without Runx2 site (*p6xNone*), or either one of the Runx2 sites (*p6xTgtggg*; *p6xTgtggc*) upstream of the *Col10a1* basal promoter and the *LacZ* gene (*βgeo*) were as illustrated (see also Table 2). Red square = TGTGG*G*; green square = TGTGG*C*; XBP = *Col10a1* basal promoter. (*B*) Reporter plasmids *pXbpβgeo*, *p6xNone*, *p-6xTgtggg*, *p-6xTgtggc*, and *Tg-4x150* were transfected into hypertrophic MCT cells. The results demonstrated that the reporter activity of plasmids *Tg-4x150*, *p-6xTgtggg*, and *p-6xTgtggc* was upregulated twofold to threefold compared to that of the reporter plasmid *pXbpβgeo*, or *p6xNone*. *pβgeo* was used as an empty vector control. A luciferase expression plasmid was cotransfected as an internal control for transfection efficiency. Bars represent the average ratios of *β*-galactosidase to luciferase activity. The standard deviations are indicated by the error bars. *p < 0.05. (*C*) Previous transgenic constructs with or without the putative Runx2 sites from the 150-bp *Col10a1* promoter (*Tg-4x150*, *Tg-6xAP*, and *Tg-6xAP^−^*)^(16)^ and the new reporter construct that uses the 83-bp 5′-sequence to drive the *LacZ* gene (*Tg-6x83*) were as illustrated. PCR genotyping showed that five mice are *Tg-6x83* transgenic founders (highlighted numbers). Both transgenic founders and wild-type littermates showed only nonspecific blue staining in the limbs and ribs at P1 stage by whole-mount X-gal staining (white arrows). P/N = PCR controls; WT = wild-type. (*D*) Histological analysis confirmed that sagittal sections of proximal femur from *Tg-6x83* transgenic mice are negative for X-gal staining (right) compared to the *Tg-6xAP^−^* transgenic mice that show reporter expression throughout the hypertrophic zone (left).^(16)^ The wild-type littermates showed no staining (data not shown). *Tg* = transgenic mice; HZ = hypertrophic zone.

MCT cells were grown at 32°C in standard DMEM with 8% fetal bovine serum (FBS; Gibco BRL) and 8% CO_2_ as per published protocol.^(15, 20)^ Following reporter constructs with cis elements derived from the 150-bp *Col10a1* distal promoter (−4296 to −4147 bp) and its basal promoter (xbp, −220 to +110 bp) driving the *LacZ* gene were generated for in vitro transfection studies. These reporter constructs are on a *pSA-βgeo-bpA* vector backbone as previously described.^(15,16, 21)^ Specifically, reporter construct *pxbp-βgeo* contains only *Col10a1* basal promoter and the *LacZ* gene. Reporter constructs containing six copies of the cis elements without the Runx2 site (*p6xNone-xbp-βgeo*, −4284 to −4232 bp), or with either one of the tandem-repeat Runx2 binding sites (*p6xTgtggg-xbp-βgeo*, −4234 to −4182 bp and *p6xTgtggc-xbp-βgeo*, −4184 to −4132 bp) were as illustrated (Fig. 3*A*; Table 2). The DNA fragments were obtained by annealing the commercially synthesized oligos (IDT Technologies). The BamHI and BglII adapters at either end were used for generation of multiple copies of the cis elements and only in the forward direction (Table 2). The reporter plasmids as well as the empty vector control (*βgeo*) or the *pRSVluc* luciferase expression plasmid for transfection efficiency were transfected into hypertrophic MCT cells as previously described.^(15, 16)^ Luciferase and *β*-galactosidase activity assays were measured using D-luciferin and the chemiluminescent assay kit (Tropix, Bedford, MA, USA).^(22)^ Transfection was performed in triplicate at three doses to ensure a linear dose-response.

**Table 2:** Oligo and Primer Sequences Used for Reporter Constructs

Reporter construct	Annealing oligos and primer sequences
*p6xNone*-*Fwd* (−4284 to −4232 bp)	5′-*GATCC*ACGTAGAATAAGCTCCTTCATAAAGTCACAGACCAGTCAGGCTGAACAGCTCC*A*-3′ (*GATCC/A*: *BamH I / Bgl II* adapter)
*p6xNone*-*Rev*	5′-*GATCT*GGAGCTGTTCAGCCTGACTGGTCTGTGACTTTATGAAGGAGCTTATTCTACGT*G*-3′ (*GATCT / G*: *Bgl II / BamH I* adapter)
*p6xTgtggg*-*Fwd* (−4234 to −4182 bp)	5′-*GATCC*TCCGAGGAAACACCCAGAATAAAAATAGTTTAATACACACAATTAGG *TGTGGG A*-3′ (*GATCC / A*: *BamH I / Bgl II* adapter, *TGTGGG*: putative Runx2 binding site)
*p6xTgtggg-Rev*	5′-*GATCT*CCCACACCTAATTGTGTGTATTAAACTATTTTTATTCTGGGTGTTTCCTCGGAG-3′ (*GATCT / G*: *Bgl II / BamH I* adapter)
*p6xTgtggc-Fwd* (−4184 to −4132 bp)	5′-*GATCC*GGG *TGTGGC* CAGCAAATACTCTGATTCTACAATCTGTTTTGGACAGGGCAGTG*A*-3′ (*GATCC / A*: *BamH I / Bgl II* adapter, *TGTGGC*: putative Runx2 binding site)
*p6xTgtggc*-*Rev*	5′-*GATCT*CACTGCCCTGTCCAAAACAGATTGTAGAATCAGAGTATTTGCTGGCCACACCC*G*-3′ (*GATCT / G*: *Bgl II / BamH I* adapter)
*Tg-6x83-Fwd* (−4296 to −4214 bp)	5′-AAT*GGATCC*TCCTGTTTCACGTAG-3′ (*GGATCC*: *BamH I* linker)
*Tg-6x83-Rev*	5′-TTT*AGATCT*ATTCTGGGTGTTTCC-3′ (*AGATCT*: *Bgl II* linker)
*Tg-6x88-Fwd* (−4234 to −4147 bp)	5′-ACA*GGATCC*GAGGAAACACC-3′ (*GGATCC*: *BamH I* linker)
*Tg-6x88-Rev*	5′-AAC*AGATCT*GTAGAATCAGAG-3′ (*AGATCT*: *Bgl II* linker)
*Tg-12xLP3-Fwd* (−4201 to −4163 bp)	5′-*GATCC*TACACACAATTAGGTGTGGGTGTGGCCAGCAAATACTCT*A*-3′ (*GATCC / A*: *BamH I / Bgl II* adapter)
*Tg-12xLP3-Rev*	5′-*GATCT*AGAGTATTTGCTGGCCACACCCACACCTAATTGTGTGTAG*G*-3′ (*GATCT / G*: *Bgl II / BamH I* adapter)

Box = putative Runx2 binding sites; *GATCC*/*A* = BamH I/Bgl II adapter.

### Transgenic reporter constructs

**Figure 4:**
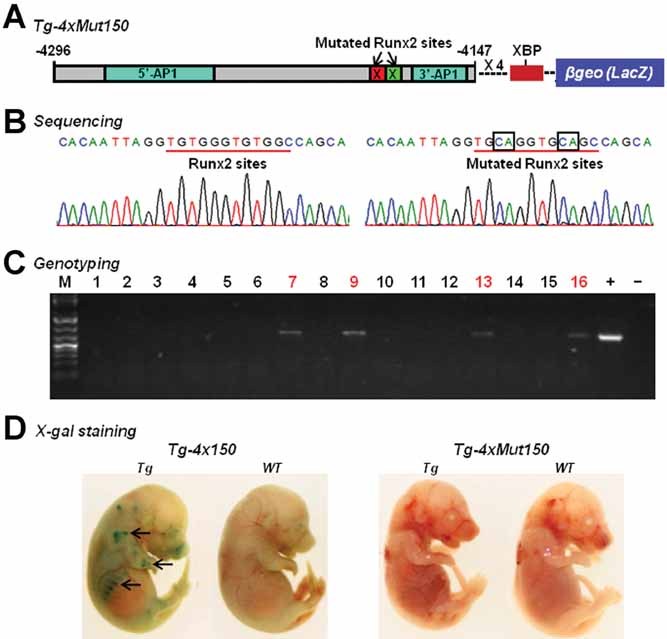
Putative Runx2 sites are required for *Col10a1* promoter activity in vivo. (*A*) Reporter construct was generated using the same 150-bp *Col10a1* distal promoter with introduced mutations in the two Runx2 binding sites (four copies) upstream of the *Col10a1* basal promoter to drive the *LacZ* gene (*Tg-4xMut150*). Red square with an “X” = TGTGG*G* replaced by TG*CA*G*G*; Green square with an “X” = TGTGG*C* replaced by TG*CA*G*C*; XBP = *Col10a1* basal promoter. (*B*) Sequencing of the transgenic reporter construct *Tg-4xMut150* confirmed that the putative Runx2 sites TGTGG*G*-TGTGG*C* have been mutated into TG*CA*G*G*-TG*CA*G*C*, the same mutations as designed for the EMSA studies (Fig. 1*C*). (*C*) PCR genotyping using *LacZ*-specific primers showed that we have generated four transgenic founder mice (highlighted numbers). M = DNA marker; “+” and “−” are PCR controls. (*D*) Whole mount X-gal staining of transgenic mice at the stage of E15.5 from previous transgenic mouse line (*Tg-4x150*) showed specific blue staining in the chondro-osseous junctions of the limbs and ribs (left panel, arrows). Histological analysis confirmed that the blue staining is within the hypertrophic zone as previously reported.^(16)^ Neither the four transgenic founders nor the wild-type littermates show reporter expression (blue staining indicating *β*-galactosidase activity) in the newly generated transgenic mouse line (*Tg-4xMut150*) with mutations inside the Runx2 binding core sequence (right panel shows representative transgenic founder and wild-type littermate,). *WT* = wild-type littermates; *Tg* = transgenic mice.

**Figure 5:**
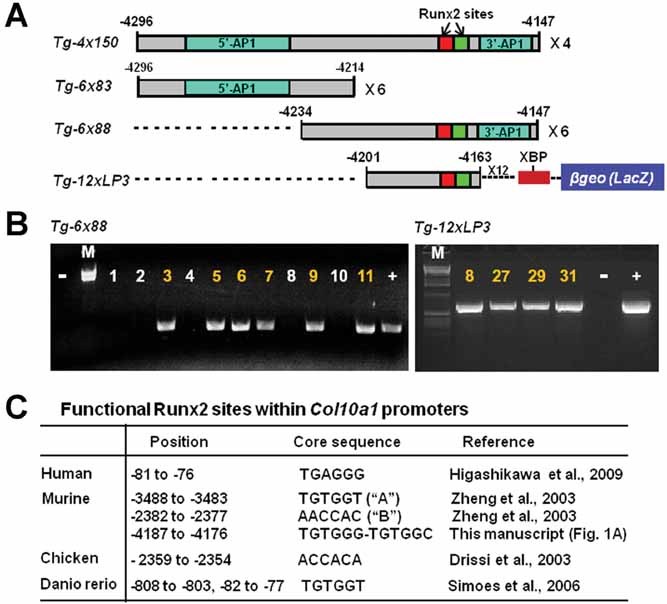
Additional elements/factors are required for *Col10a1* promoter activity. (*A*) Two additional transgenic constructs using the 3′-sequence of the 150-bp *Col10a1* distal promoter to drive the *LacZ* gene were illustrated and compared to the ones using the 5′-sequence or the whole 150-bp promoter.^(16)^ Reporter construct *Tg-6x88* contains six copies of the 3′-sequence (−4234 to −4147 bp, top), while transgenic construct *Tg-12xLP3* contains 12 copies of the 3′-sequence (−4201 to −4163 bp, top) upstream of the same *Col10a1* basal promoter and the *LacZ* gene. Both constructs contain the tandem-repeat Runx2 binding sites. XBP = *Col10a1* basal promoter. (*B*) PCR genotyping using *LacZ*-specific primers showed that five mice, numbered 3, 5, 6, 7, 9, and 11, are *Tg-6x88* transgenic founders (left). +/− = positive/negative PCR control. These founder mice were sacrificed at P1 stage and subjected to whole-mount X-gal staining. No cell-specific blue staining was observed in either these transgenic founders or their littermate controls (data not shown). Four mouse lines from *Tg-12xLP3* transgenic founders (numbered 8, 27, 29, and 31; right) were established and subject to reporter (*LacZ*) analysis. Neither of these transgenic mouse lines at the P1 stage show blue staining in hypertrophic chondrocytes (data not shown). (*C*) Listed are the putative Runx2 binding sites within the human, murine, chicken, and zebrafish (*Danio rerio*) type X collagen gene promoters. The positions of Runx2 sites are found to be located in the basal (human, −81 to −76 bp; zebrafish, −82 to −77 bp), proximal (murine, −3488 to −3483 bp, −2382 to −2377 bp; zebrafish, −808 to −803 bp), or distal promoters (murine, −4187 to −4176 bp; chicken, −2359 to −2354 bp) of the type X collagen gene. The core binding sequence of Runx2 sites also varies within or among the different species, including TGAGGG, TGTGGT, AACCAC, ACCACA, TGTGGG-TGTGGC, etc. The sources describing the putative function of correspondent Runx2 sites are also listed: Higashikawa and colleagues,^(7)^ Zheng and colleagues,^(15)^ Drissi and colleagues,^(24)^ and Simões and colleagues.^(26)^

Transgenic reporter constructs containing 83 bp of the 5′-sequence (*Tg-6x83*, −4296 to −4214 bp, six copies; Fig. 3*C*) of the 150-bp fragment were generated by PCR amplification. The oligos were designed with BamHI or BglII linkers added to the 5′ or 3′ end respectively, such that multiple copies can be generated by enzyme digestion followed by linear ligation (Fig. 3*C*; Table 2). A reporter construct containing the same 150-bp *Col10a1* distal promoter with introduced mutations inside the Runx2 binding core sequence was also generated by PCR amplification (*Tg-4xMut150*, four copies; Fig. 4*A*). The forward primer, 5′-AAT *GGA TCC* TCC TGT TTC ACG TAG-3′, has a BamHI linker at the 5′-end (italic) and has been reported previously.^(16)^ The reverse primer, 5′-AAC *AGA TCT* GTA GAA TCA GAG TAT TTG CTG GCT GCA CCT GCA CCT AAT TGT GTG-3′, has a BglII linker at the 5′-end (italic) and was designed with the same mutations as probes *SP9* and *LP3* that were used in the EMSA studies (underlined, Fig. 1*A*, *C*). The PCR product containing the designated mutations was subjected to sequencing confirmation (Fig. 4*B*) using the DNA Service Facility (DNAS) at the University of Illinois at Chicago (UIC). Transgenic reporter constructs containing 88 bp of the 3′-sequence (*Tg-6x88*, −4234 to −4147 bp, six copies; Fig. 5*A*) of the 150-bp fragment were also generated by PCR amplification. Transgenic construct containing the sequence of *LP3* (*Tg-12xLP3*, −4201 to −4163 bp, 12 copies) was generated by annealing the complementary oligos synthesized by IDT Technologies (Fig. 5 *A*; Table 2). Multiple copies of these 39-bp, 83-bp, 88-bp, and 150-bp (with mutations) *Col10a1* promoter fragments were placed upstream of the same *Col10a1* basal promoter (−220 to +110 bp) and the *LacZ* gene as described.^(16)^

### Transgenic mouse studies

The transgenic cassette containing *Col10a1* promoter elements and the *LacZ* gene were released by *Not I* and *Sal I* digestion followed by QIXII purification (Qiagen). Purified DNA was microinjected into fertilized mouse eggs and implanted into FVB pseudopregnant foster mothers using the Axiovert 200 transgenic apparatus (Carl Zeiss, Germany) or conducted at the University of Illinois at Chicago (UIC) Transgenic Production Service core facility.^(15)^, ^(16)^ PCR genotyping was performed using *LacZ*-specific primers. The transgenic founder mice at embryonic day 15.5 (E15.5) were subjected to whole-mount X-gal staining as previously described.^(15, 16)^ Mice at the postnatal day 1 (P1) stage were X-gal stained, paraffin embedded, sectioned, and counterstained with nuclear fast red (Poly Scientific R&D Corp.) to examine *LacZ*/reporter expression.^(15, 16)^ Sagittal sections of the limb growth plate from both transgenic and wild-type littermates were analyzed using a Nikon microscope (Nikon Eclipse 80i; Nikon Instruments Inc., Melville, NY, USA) and the Qcapture Suite software (version 2.95.0; Quantitative Imaging Corp., USA). At least 30 sections of each growth plate were analyzed. The animal studies were approved by the animal care and oversight committees at Baylor College of Medicine, University of Illinois at Chicago and Rush University Medical Center.

## Results

### Putative transcription factors bind the 3′-sequence of the 150-bp *Col10a1* cis-enhancer

We have previously shown that a 150-bp *Col10a1* distal promoter (−4296 to −4147 bp) is sufficient to direct hypertrophic chondrocyte-specific reporter expression in vivo.^(16)^ To further localize the cis enhancer element and to identify its binding factors, we have performed EMSAs using a series of annealed DNA oligomers derived from this 150-bp promoter and the hypertrophic MCT cell nuclear extracts. These oligos include 11 short consecutive and overlapping oligomers (termed short probe, *SP*) and three longer oligomers (termed long probe, *LP*) that cover the 150-bp *Col10a1* promoter region (Fig. 1*A*; Table 1). MCT cells are a cell model of chondrocyte hypertrophy that show enhanced *Col10a1* expression upon growth arrest as previously described.^(15,16, 20)^ We observed two specific DNA/protein complexes that formed with *SP9* with different signal intensity (Fig. 1*B*, lane 9). Similar but more intense signals for the DNA/protein complexes were observed when *LP3* was used compared to *SP9* (Fig. 1*B*, lane 9 and lane *LP3*). *LP3* (−4201 to −4163 bp) covers the entire sequence of *SP9* (−4196 to −4172 bp; Fig. 1*A* and Table 1). A weak DNA/protein complex was also observed when *SP11*, which ranges from −4171 to −4147 bp, was used (Fig. 1*B*, lane 11). These results suggest that certain transcription factors which upregulate *Col10a1* expression in hypertrophic MCT cells directly interact with an approximately 30-bp sequence (−4200 to −4170 bp) located at the 3′-end of the 150-bp *Col10a1* promoter region.

### Putative Runx2 sites are core sequences to form the specific DNA/protein complex

We have performed detailed sequence analysis of this short 30-bp sequence and identified two putative tandem-repeat Runx2 binding sites (TGTGG***G***-TGTGG***C***, −4187 to −4176 bp; Fig. 1*A*) based on our previous studies and literature review.^(15, 23)^ To study if these putative Runx2 sites are the core binding sequence responsible for formation of the specific DNA/protein complexes, we have performed similar EMSA assays using *LP3* and *SP9* as well as their mutant derivatives. The mutant probes contain mutations inside (MI) or outside (MO) of the putative Runx2 core binding sites (Fig. 1*C*). The results showed that mutations inside the core binding sequence of both *LP3* and *SP9* abolished the binding complexes (Fig. 1*D*; MI, lane 1) that formed when wild-type probes were used (Fig. 1*D*; WT, lane 1). Meanwhile, specific DNA/protein complexes were still observed when probes containing mutations outside the core binding sequence were used (Fig. 1*D*; MO, lane 1). No DNA/protein complexes were seen in lanes that used no biotin-labeled competitive probes (Fig. 1*D*, lane 2). These data suggest that the two putative Runx2 binding sites are the core binding sequence required for both *LP3* and *SP9* to form specific DNA/protein complexes with hypertrophic MCT cell nuclear extracts.

### Runx2 interacts with the putative Runx2 binding sites in vitro

We have performed candidate EMSA assays using the same biotin-labeled (Bio) *LP3* and *SP9* as well as Runx2 antibody (R2Ab). As shown in Figure 2*A*, decreased signal intensity of both complexes was observed in lanes that used Runx2 antibody (Fig. 2*A*, “R2Ab” lanes, dashed arrows) compared to the one which used only biotin-labeled *LP3* and *SP9* (Fig. 2*A*, “Bio” lanes, black arrows). No signal was shown in corresponding lanes when competitive probes (without biotin-labeling) were added (Fig. 2*A*, “Comp” lanes). The results suggest that Runx2 antibody inhibits the binding of the putative Runx2 sites with the hypertrophic MCT cell nuclear extracts. To investigate if Runx2 antibody dose-dependently inhibits formation of the binding complexes by MCT cell nuclear extracts and the putative Runx2 sites, we have performed quantitative candidate EMSA assays with both probe *LP3* and probe *SP9* using a various doses of the Runx2 antibody (sc-8566; Santa Cruz Biotechnologies) at 200, 500 and 1000 ng, respectively. As illustrated in Figure 2*B*, obvious signal reduction was observed when 200 ng of Runx2 antibody was used. Meanwhile, increasing the amount of Runx2 antibody to 500 ng further inhibited the binding activity of the putative Runx2 sites with the MCT cell nuclear extracts, while 1000 ng of Runx2 antibody completely abolished formation of the DNA/protein complexes for both *LP3* and *SP9*. In addition, we repeated the dose-response experiment using *SP9* and Runx2 antibody in parallel with a control antibody, anti-Hif1α (sc-13515). We only observed significant signal reduction with Runx2 antibody, not with anti-Hif1α (Supplemental Fig. S1). These results demonstrate that Runx2 antibody specifically inhibits the binding activity of the putative Runx2 sites with hypertrophic MCT cell nuclear extracts. Runx2 is one of the components within hypertrophic MCT cell nuclear extracts that form the specific DNA/protein complexes.

### Runx2 interacts with the *Col10a1* distal promoter in MCT cells

A chromatin immunoprecipitation (ChIP) assay using the Runx2 antibody was performed to affirm the binding of Runx2 and the putative Runx2 sites in MCT cells. As illustrated in Figure 2*C*, we performed semiquantitative PCR with the primers flanking the putative Runx2 sites or control sequence (Fig. 2*C*, top, arrows) using DNA immunoprecipitated by Runx2 antibody or control IgG. We observed clear amplicon from the enhancer region that used Runx2 antibody (Fig. 2*C*, left, lane R2Ab), whereas DNA precipitants from control IgG only showed a faint band (Fig. 2*C*, left, lane IgG). Meanwhile, PCR amplicon was barely detectable from the control sequence that used either Runx2 antibody (Fig. 2*C*, right, lane R2Ab) or control IgG (Fig. 2*C*, right, lane IgG). PCR amplicons were obtained from the nonprecipitated input sample both for target enhancer and control sequence (Fig. 2*C*, “Input” lanes). We have also performed quantitative PCR using the same DNA templates and the same pairs of primers flanking the putative Runx2 sites or control sequence. The result showed that the target enhancer sequence containing the putative Runx2 sites was significantly enriched (7.4-fold) from a given amount of input (5–10 µg chromatin) by Runx2 antibody compared to the one using control IgG (Fig. 2*D*, Enh-IgG, 1.000/0.029, Enh-R2Ab, 7.438/0.873; *p* = 0.009, *n* = 2). While the control sequence without putative Runx2 sites does not show any enrichment from the same amount of input sample by Runx2 antibody (Fig. 2*D*, Ctrl-IgG, 1.030/0.346, Ctrl-R2Ab, 1.115/0.597; *p* = 0.878, *n* = 2). These results together suggest that Runx2 binds to the 150-bp *Col10a1* distal promoter containing the putative Runx2 binding sites in MCT cells.

### Putative Runx2 binding sites mediate upregulated reporter activity in vitro

To determine if the putative Runx2 binding sites within the 150-bp *Col10a1* distal promoter mediate upregulated *Col10a1*/reporter activity in vitro, we performed transfection studies using hypertrophic MCT cells and reporter constructs that were derived from this promoter region. These constructs were generated with or without the Runx2 binding sites (Table 2) and are illustrated in Figure 3*A*. Briefly, reporter plasmids containing *Col10a1* basal promoter (*xbp*) and the *LacZ* gnene (*p-xbp-βgeo*), the transgenic reporter construct containing the two intact Runx2 binding sites (*Tg-4x150*), or cis-elements with either one of the Runx2 binding sites (*p-6xTgtggg-xbp-βgeo* and *p-6xTgtggc-xbp-βgeo*), or a control plasmid without a Runx2 binding site (*p6xNone-xbp-βgeo*) upstream of the *Col10a1* basal promoter and the *LacZ* gene were transfected into hypertrophic MCT cells. A RSV-luc luciferase expression plasmid was cotransfected as an internal control for transfection efficiency. The results showed that the reporter plasmid *p-6xNone-xbp-βgeo* had only basal *β*-galactosidase activity similar to that of the control plasmid *p-xbp-βgeo*, while the relative *β*-galactosidase activity of the plasmids containing either one or both Runx2 binding sites (*p-6xTgtggg-xbp-βgeo* and *p-6xTgtggc-xbp-βgeo*, and *Tg-4x150*) was upregulated by two- to threefold compared to that of plasmid *p-6xNone-xbp-βgeo* or the control plasmid *p-xbp-βgeo* (Fig. 3*B*). The empty vector *pβgeo* (*pSA-βgeo-bpA*) showed only background *β*-galactosidase activity. These results suggest that reporter constructs containing the putative Runx2 binding sites are able to mediate upregulated reporter activity in hypertrophic MCT cells.

### Transgenic studies using 5′-sequence of the 150-bp *Col10a1* distal promoter

Our previous transgenic studies have shown that the 150-bp *Col10a1* distal promoter or the same promoter with the two AP1 elements deleted was able to direct cell-specific *Col10a1*/reporter expression in vivo (Fig. 3*C*; *Tg-4x150*, *Tg-6xAP*^*−*^), while the reporter construct with the two AP1 elements did not give any tissue specificity (Fig. 3*C*, *Tg-6xAP*).^(16)^ These results localize the major cis-enhancer to either the short 5′-sequence (−4296 to −4280 bp) or the longer 3′-sequence (−4238 to −4171 bp) of the 150-bp *Col10a1* distal promoter. To determine the contribution of the 5′-sequence to *Col10a1* tissue specificity, we generated another transgenic construct using the 5′-sequence (−4296 to −4214 bp, six copies) upstream of the same *Col10a1* basal promoter to drive the *LacZ* gene (Fig. 3*C*, *Tg-6x83*). PCR genotyping using *LacZ*-specific primers showed that we generated five transgenic founders (Fig. 3*C*, bottom/left, lanes 4, 11, 12, 13, and 15). As expected, X-gal staining of these transgenic founders at P1 detected only background staining in craniofacial and bony tissues compared to their littermate controls (Fig. 3*C*, bottom/right, arrows). Histological analysis confirmed that no cell-specific X-gal staining was observed in the hypertrophic chondrocytes of the *Tg-6x83* transgenic mice (Fig. 3*D*, right) compared to that of the *Tg-6xAP*^*−*^ transgenic mice (Fig. 3*D*, left). This result suggests that the 5′-sequence of the 150-bp *Col10a1* promoter has less importance in mediating its cell-specific expression. The major cis-enhancer responsible for its promoter activity is probably within the 3′-end, which is about 43 bp (−4213 to −4171 bp), containing the tandem-repeat Runx2 binding sites.

### Transgenic studies using the 150-bp *Col10a1* distal promoter with mutated Runx2 sites

To determine the in vivo role of the Runx2 sites in the context of the 150-bp *Col10a1* distal promoter that has been shown to confer hypertrophic chondrocyte-specific reporter activity,^(16)^ we have generated a new transgenic construct. In this new construct, we used the same 150-bp distal promoter (with the two inside Runx2 sites mutated) upstream of the *Col10a1* basal promoter to drive the *LacZ* gene (*Tg-4xMut150*; Fig. 4*A*). The two Runx2 binding sites have been mutated according to the mutations used in the EMSA studies and were confirmed by sequencing (Fig. 4*B*). We have successfully generated four transgenic founders as confirmed by PCR genotyping using *LacZ*-specific primers (Fig. 4*C*).

All these transgenic founders and their wild-type littermates at E15.5 were subjected to the same X-gal staining protocol as previously described to show reporter (*lacZ*) activity.^(15)^, ^(16)^ Neither of the founders showed blue staining in the long bones or ribs (Fig. 4*D*, right panel), while previous transgenic studies (*Tg-4x150*) showed that the same 150-bp *Col10a1* promoter containing intact Runx2 sites directs specific reporter expression in hypertrophic zone (the chondro-osseous junctions of the limbs and ribs; Fig. 4*D*, left panel).^(16)^ Together, these results further indicated the importance of the 3′-sequence containing the tandem-repeat Runx2 binding sites in mediating cell-specific *Col10a1* promoter activity in vivo.

### Transgenic studies using the 3′-sequence of the *Col10a1* distal promoter

The combined results from in vitro binding and in vivo transgenic reporter analyses suggest that the major *Col10a1* cis-enhancer is localized to a 43-bp 3′-sequence containing the putative Runx2 sites (Figs. 1–4).^(16)^ To directly determine the in vivo relevance of this putative enhancer, we used a similar strategy to generate a transgenic reporter construct with the 3′-sequence (−4234 to −4147 bp, six copies) to drive the *LacZ* gene (Fig. 5*A*, *Tg-6x88*). This construct has a 20-base overlapping sequence with the 5′-sequence (*Tg-6x83*; Fig. 3*C*, Fig. 5*A*). As illustrated, we successfully generated six transgenic founders based on PCR genotyping (Fig. 5*B*, bottom/left, lanes 3, 5, 6, 7, 9, and 11). Surprisingly, X-gal staining and histological analysis of the *Tg-6x88* transgenic founders at the P1 stage did not show cell-specific blue staining in the hypertrophic chondrocytes (data not shown). We have also generated an additional transgenic construct using more copies of the *LP3* sequence that contains the tandem-repeat Runx2 sites (Fig. 1*A*, C; Table 1, −4201 to −4163 bp, 12 copies) to drive the *LacZ* gene. PCR genotyping confirmed that we have generated four transgenic founders (Fig. 5*B*, bottom/right, lanes 8, 27, 29, and 31, *Tg-12xLP3*). We have examined reporter (*LacZ*) activity for each of these transgenic mouse lines and no blue staining indicating *β*-galactosidase activity was observed in the hypertrophic chondrocytes of these transgenic mice (data not shown). These results, together with previous transgenic studies (Fig. 3*C*, D; Fig. 4),^(16)^ suggest that the 3′-sequence containing the Runx2 binding sites is required but not sufficient to direct cell-specific *Col10a1*/reporter expression in vivo^(15, 16, 20)^ Additional cooperative or repressive elements within the 5′- or 3′-sequence are also required for cell-specific *Col10a1* promoter activity.

### Multiple Runx2 binding sites within the type X collagen gene promoters

Through detailed literature review, we summarize here the multiple putative Runx2 binding sites that have recently been reported within human, murine, chicken, and zebrafish (*Danio rerio*) type X collagen gene promoters (Fig. 5*C*). These Runx2 sites are distributed throughout the proximal and distal promoters of the *Col10a1* gene and have been shown to contribute to cell-specific *Col10a1* expression both in vitro and in vivo.^(7, 15)^, 24–26 There are differences within the Runx2 core binding sequence, including TGAGGG (human),^(7)^ TGTGGT (murine and zebrafish),^(15, 26)^ AACCAC (murine),^(15)^ ACCACA (chicken),^(24, 25)^ and the novel tandem-repeat sites TGTGGG-TGTGGC as described (Fig. 5*C*). These studies together demonstrate that Runx2 is a key player and may work through different mechanisms, ie, interact with variant core binding sequences and the cofactors, to regulate cell-specific type X collagen gene expression.

## Discussion

In recent years, significant progress has been made toward characterization of the cell-specific type X collagen gene regulation during chondrocyte hypertrophy or maturation. Several putative transcription factors (such as Sp1, AP1, Runx2, MEF2C, Hif-2a, etc.) have been shown to interact with multiple cis elements of zebrafish, chicken, murine, and human *Col10a1* promoters. These interactions are known to contribute to tissue-specific type X collagen gene expression.^(7, 8, 15)^, 27–34 However, to date, the refined cis-enhancer within the murine *Col10a1* promoter has not been fully characterized. Candidate transcription factors that are responsible for murine *Col10a1* expression are therefore, still not well defined. In this work, we report further characterization of the 150-bp *Col10a1* distal promoter or enhancer element (−4296 to −4147 bp) that has been shown to direct hypertrophic chondrocyte-specific reporter expression in vivo.^(16)^

We performed EMSA using DNA oligomers derived from this 150-bp *Col10a1* promoter and the hypertrophic MCT cell nuclear extracts. We only observed clear and specific DNA/protein complexes with an approximately 30-bp sequence located within its 3′-end (−4200 to −4170 bp; Fig. 1*A*, *B*). As previously described, MCT cells express type X collagen abundantly upon growth arrest.^(15, 20)^ Therefore, transcription factors that form specific binding complexes with this putative *Col10a1* cis-enhancer element are very likely responsible for upregulated *Col10a1* expression in hypertrophic MCT cells. Interestingly, we identified two putative tandem-repeat Runx2 binding sites within this 30-bp sequence (TGTGG***G***-TGTGG***C***; Fig. 1*A*, −4187 to −4176 bp). These Runx2 sites were not identified by TFSearch or rVista2 due to the 1-base discrepancy with the previously reported Runx2 core binding sequence (TGTGG***T***),^(15, 26, 35, 36)^ although one of the tandem-repeat sequences, “TGTGGC,” was previously shown to bind Runx2.^(23)^

We subsequently performed EMSA studies with mutated DNA elements (Fig. 1*C*) and demonstrated that the two putative Runx2 sites constitute the core binding sequence that is required to form the specific DNA/protein complexes (Fig. 1*D*). Our quantitative and candidate EMSA assays suggest that Runx2 is one of the components of the DNA/protein binding complex(es). Increasing the concentration of Runx2 antibody resulted in decreased signal intensity of the DNA binding complexes, whereas no signal reduction was observed when the control antibody, anti-Hif1α, was used for similar dose-response experiments. We noticed that Runx2 antibody usually causes a supershifted band as demonstrated previously.^(15)^ We performed EMSA using the nonradioactive LightShift Chemiluminescent EMSA kit (Pierce). This system may not be sensitive enough to detect the signals of the shifted band, which usually represents only a very small portion of the binding complexes. Detection of signal reduction (instead of the shifted band) using this EMSA assay system was recently reported.^(37)^ Moreover, chromatin immunoprecipitation (ChIP) experiments further confirmed the specific and direct interaction between Runx2 and the putative *Col10a1* cis-enhancer containing the tandem-repeat Runx2 sites in MCT cells (Fig. 2*C*, *D*).

Our in vitro transfection studies showed that reporter constructs containing the putative Runx2 sites are able to mediate upregulated *Col10a1* promoter activity in hypertrophic MCT cells. We have also performed real-time RT-PCR analysis using total RNAs prepared from proliferative and hypertrophic MCT cells. The results showed that Runx2 is upregulated (fourfold) in hypertrophic MCT cells, whereas *Col10a1* mRNA transcript is much more abundant (more than 20-fold) in hypertrophic MCT cells than in proliferative MCT cells (Supplemental Fig. S2).

Our previous transgenic studies have shown that the 150-bp *Col10a1* distal promoter (*Tg-4x150*) and a 90-bp AP1 deletion mutant (*Tg-6xAP*^*−*^) are able to mediate cell-specific *Col10a1/*reporter expression, whereas sequences containing the AP1 elements did not give tissue-specificity (*Tg-6xAP*).^(16)^ Interestingly, both *Tg-4x150* and *Tg-6xAP*^*−*^ constructs contain the putative Runx2 binding sites whereas the *Tg-6xAP* construct does not include the Runx2 sites (Fig. 3*C*), suggesting the in vivo requirement of these Runx2 binding sites in mediating *Col10a1* cis-enhancer activity. As expected, additional transgenic studies using the 5′-sequence (*Tg-6x83*, −4296 to −4214 bp) without the Runx2 sites did not drive cell-specific reporter expression (Fig. 3*C*, *D*). Moreover, transgenic studies from four transgenic founders (Fig. 4) showed that mutating the Runx2 binding sites in the context of the 150-bp promoter region abolishes its capacity to confer the hypertrophic chondrocyte-specific expression of the *LacZ* gene that has been shown previously.^(16)^ These results further suggest that the major cis-enhancer responsible for cell-specific *Col10a1* expression in vivo is possibly within a 43-bp 3′-sequence (−4213 to −4171 bp) where the tandem-repeat Runx2 binding sites are located (Figs. 2*C*, 3*C*). However, we have generated two more transgenic constructs using the 3′-sequences containing the Runx2 sites (*Tg-6x88*, −4234 to −4147 bp and *Tg-12xLP3*, −4201 to −4163 bp) to drive the *LacZ* gene. Surprisingly, no cell-specific reporter expression was observed in these transgenic mice (Fig. 5). Although chromatin-mediated silencing of the transgene (*LacZ*) may occur in these transgenic studies, our results suggest that there are cooperative or repressive elements within the 5′- or 3′-sequence of the 150-bp *Col10a1* cis-enhancer which are also required for its tissue-specificity.

Recently, a growing body of data has shown that there are multiple putative Runx2 core binding sites within the basal, proximal, or distal part of the type X collagen gene promoters. These Runx2 sites contribute to regulation of *Col10a1* expression and affect chondrocyte maturation during skeletal development and multiple skeletal defects (Fig. 5*C*). However, no one, to date, has shown the direct interaction between Runx2 and the cis-enhancer element of *Col10a1* genes. In zebrafish, two Runx2 isoforms are involved in transactivation of *colX*α*1* in vitro via its conserved Runx2 binding sites located within its promoter.^(26)^ Meanwhile, Wnt/beta-catenin signaling has been shown to induce chondrocyte hypertrophy through activation of Runx2, which is also mediated by the Runx2 binding site found within the chicken *Col10a1* promoter.^(24, 25)^ More recently, a core element responsive to RUNX-2, termed the hypertrophy box (HY box), was found to be located in the human *COL10A1* proximal promoter (between −89 and −60 bp). This cis-element mediates upregulated human *COL10A1* promoter activity by reporter assays in human cells.^(7)^ As for murine *Col10a1* gene regulation, we have reported identification of conserved Runx2 binding sites within the *Col10a1* proximal promoter that can only direct weak reporter expression in lower hypertrophic chondrocytes.^(15)^

In this work, we have for the first time reported identification of the tandem-repeat Runx2 core binding sites (TGTGGG-TGTGGC) within the 150-bp *Col10a1* distal promoter/enhancer region. Interestingly, this site was previously shown to be highly conserved between human and murine type X collagen gene enhancers (Supplemental Fig. S3).^(32)^ Our data provide direct evidence that Runx2 interacts with this *Col10a1* cis-enhancer via its tandem-repeat Runx2 binding sites. Reporter constructs containing these putative Runx2 sites are able to mediate upregulated reporter activity in hypertrophic MCT cells. These putative Runx2 sites are required for cell-specific *Col10a1*/reporter expression in vivo as demonstrated by a series of transgenic reporter analyses with reporter constructs with or without the Runx2 binding sites (Figs. 3 and 4). Runx2 is a known master transcription factor for osteoblast differentiation as well as a critical regulator for chondrocyte maturation.38–43 Runx2 regulation of cell-specific *Col10a1* expression may impact the process of chondrocyte maturation and constitute the major mechanistic basis of multiple skeletal diseases, such as cleidocranial dysplasia, fracture healing, and osteoarthritis.^(7)^, 44–46 As demonstrated in previous studies and studies summarized in Figure 5*C*, multiple cis-elements may work together with Runx2 and/or its cofactors to mediate cell-specific type X collagen gene expression both in vitro and in vivo.^(7, 8, 15)^, 26–34 Our data suggest that in addition to the conserved Runx2 binding sites located within its 3′-end, there are cooperative or repressive elements^(16)^ within the 5′- or 3′-sequence of the 150-bp *Col10a1* cis-enhancer. This indicates a diversified mechanism of Runx2 regulation of type X collagen gene expression among different species, and therefore, provides important information for investigators in the fields of bone and cartilage, especially the field of growth plate biology. Further characterization of this Runx2-*Col10a1* interaction has the potential to identify novel therapeutic targets for multiple skeletal diseases showing altered *Col10a1* expression and chondrocyte maturation.

## Disclosures

All the authors state that they have no conflicts of interest.
