# Supplementary figures and images for: Runx2 contributes to murine Col10a1 gene regulation through direct interaction with its cis-enhancer
Source: J Bone Miner Res. 2011 Sep;26(12):2899–910. doi: 10.1002/jbmr.504 (PMC3222790; doi:10.1002/jbmr.504)

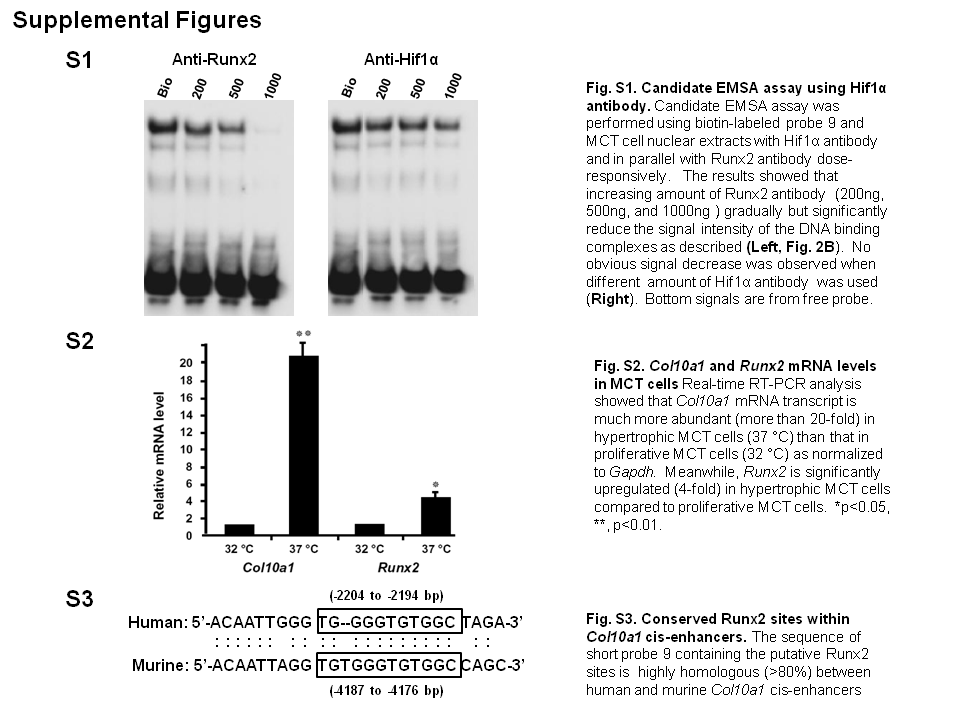

Supplement: Supplementary file 1 [file jbmr0026-2899-sd1.tif]
